# Investigating working memory and brain activation in major depressive disorder with and without insomnia: insights from functional near-infrared spectroscopy (fNIRS)

**DOI:** 10.1186/s12888-026-08471-6

**Published:** 2026-08-19

**Authors:** Yanli Li, Chao Zheng, Yanni Li, Yubo Wang, Zichen Ding, Miaomiao Xu, Tingting Li, Lei Wang, Leyu Jia, Yuanyuan Zhang, Zhiwang Liu, Lingjiang Liu, Xingxing Li, Yongming Xu, Haihang Yu, Dongsheng Zhou

**Affiliations:** 1 Department of Psychology, Affiliated Kangning Hospital of Ningbo University（Ningbo Kangning Hospital）, Ningbo Key Laboratory for Physical Diagnosis and Treatment of Mental and Psychological Disorders, Ningbo, Zhejiang, China; 2 Key Laboratory for Physical Diagnosis and Treatment of Mental and Psychological Disorders, Ningbo Kangning Hospital, 1 Zhuangyu Road, Zhejiang, China

**Keywords:** Major depressive disorder, Insomnia, Functional near-infrared spectroscopy (fNIRS), Working memory, Prefrontal cortex

## Abstract

**Background:**

Major depressive disorder (MDD) frequently coexists with insomnia, a comorbidity that exacerbates cognitive deficits, particularly in working memory (WM). While previous research has established links between insomnia and impaired brain function, the barin functional indicators associated with WM deficits in MDD patients with insomnia remain unclear. This study explores cortical activation patterns in MDD patients with and without insomnia using functional near-infrared spectroscopy (fNIRS).

**Methods:**

A total of 55 MDD patients with insomnia and 67 without insomnia were recruited. Cortical activation during WM tasks was assessed using fNIRS, focusing on oxygenated hemoglobin (Oxy-Hb) concentration changes. Sleep quality was assessed using the Pittsburgh Sleep Quality Index (PSQI), depression severity was assessed using the Hamilton Depression Scale (HAMD), and cognitive function was assessed using the Repeatable Battery for the Assessment of Neuropsychological Status (RBANS).

**Results:**

MDD patients with insomnia exhibited lower RBANS scores and reduced accuracy in medium-load WM tasks compared to non-insomnia patients. Additionally, fNIRS analysis revealed diminished Oxy-Hb concentrations in the bilateral dorsolateral prefrontal cortex (DLPFC) during medium-load tasks and in the left DLPFC during high-load tasks. Correlation analyses indicated that immediate memory scores negatively correlated with left DLPFC and bilateral medial prefrontal cortex (mPFC) activation during medium-load tasks, while task accuracy negatively correlated with bilateral mPFC activation (*p* < 0.05).

**Conclusion:**

The presence of insomnia in MDD is associated with exacerbated WM deficits and altered prefrontal cortical activation, particularly in tasks with increased cognitive demand. These findings provide potential neurofunctional reference indicators, which may help offer auxiliary assessment means for MDD patients with insomnia. In addition, the sensitivity of fNIRS in detecting these neural brain activation differences suggest it may have potential as an exploratory screening tool for identifying subtypes of depression.

**Supplementary Information:**

The online version contains supplementary material available at https://doi.org/10.1186/s12888-026-08471-6.

## Introduction

Major Depressive Disorder (MDD) is a leading global psychiatric disorder, affecting over 322 million people worldwide. It is projected to become the primary cause of disease burden within the next two decades. Core symptoms of MDD include persistent low mood, anhedonia, fatigue, cognitive impairments (e.g., reduced attention and low self-esteem), and sleep disturbances [1, 2]. Notably, insomnia affects 67–84% of MDD patients during depressive episodes, exacerbating symptom severity, impairing recovery, and increasing risks of cardiovascular and autoimmune comorbidities [3–5]. While insomnia’s role in emotional dysregulation is well-documented, its contribution to cognitive dysfunction—particularly working memory deficits—remains poorly understood, despite evidence linking insomnia severity to poorer task accuracy and slower processing speeds in MDD patients [6, 7].

Cognitive impairments, especially working memory deficits, are a hallmark of MDD and critically predict functional disability and relapse risk [8]. These deficits may represent endophenotypes with distinct pathophysiology, yet current clinical practices prioritize emotional symptom management, overlooking cognitive dysfunction as a secondary concern [9]. To address this gap, functional near-infrared spectroscopy (fNIRS) offers a unique tool for mapping cortical hemodynamics in clinical settings. Unlike fMRI, fNIRS combines portability with tolerance to movement artifacts, enabling real-time monitoring of prefrontal activation during ecologically valid tasks [10]. Prior fNIRS studies have revealed reduced dorsolateral prefrontal cortex (DLPFC) activation in MDD patients during working memory tasks [11, 12]. However, no studies have compared activation patterns between MDD patients with and without insomnia—a critical omission given insomnia’s potential to affect prefrontal function.

This study hypothesizes that MDD patients with insomnia will exhibit (1) poorer working memory performance, (2) reduced DLPFC activation during medium/high-load tasks, and (3) a stronger correlation between DLPFC hypoactivation and cognitive deficits in the insomnia subgroup. By elucidating these neural functional differences, our research aims to explore potential neural functional indicators and to develop exploratory screening methods for MDD patients with comorbid insomnia.

## Methods

### Subjects

This study was conducted at Kangning Hospital in Ningbo, Zhejiang Province, from July 2021 to December 2023 and included 122 eligible patients diagnosed with MDD. Participants were recruited through physician referrals, with doctors introducing the research project to potential candidates. Inclusion criteria required patients to meet the diagnostic criteria for MDD as defined by the (Inclusion criteria for subjects included:) 1) Diagnostic and Statistical Manual of Mental Disorders, Fifth Edition (DSM-5), 2) age between 16 and 65 years, and have a Hamilton Depression Rating Scale-24 (HAMD-24) total score of ≥20, indicating moderate to severe depression. Exclusion criteria included a history of psychosis, significant physical or organic brain diseases, substance abuse or dependence (except for cigarette smoking), and pregnancy or lactation.

Prior to the study’s commencement, ethical approval was obtained from the Ethics Committee of Ningbo Kangning Hospital (NBKNYY-2023-LC-31), ensuring compliance with the ethical standards and regulations of the Declaration of Helsinki on Human Research. All participants were provided with a comprehensive explanation of the study’s purpose, procedures, and potential risks. Those who agreed to participate voluntarily provided written informed consent, and minor participants have voluntarily provided written informed consent through their legal guardians. These steps ensured that the study adhered to ethical standards and that participants were fully informed about their involvement in the research.

### Measures

In this study, depressive symptoms in patients with MDD were assessed using the HAMD-24. The total HAMD-24 score reflects the severity of depression, with scores interpreted as follows: ≤7 indicates no depressive symptoms, 8–20 suggests probable depression, 20–35 indicates definite depression, and >35 signifies severe depression. Higher scores correspond to more severe depressive symptoms [13]. Sleep quality was evaluated using the Pittsburgh Sleep Quality Index (PSQI), a seven-component scale that assesses sleep quality, latency, duration, efficiency, disturbances, use of hypnotics, and daytime dysfunction over the past month. Each component is scored from 0 to 3, and the cumulative score ranges from 0 to 21, with higher scores indicating poorer sleep quality [14]. In this study, insomnia was defined as a total PSQI score of ≥11.

Immediate Memory (IM) was assessed using items 1 (word learning) and 2 (story retelling) from the Repeatable Battery for the Assessment of Neuropsychological Status (RBANS). Raw scores were converted to scaled scores based on American norms, and the two scores were combined to generate an immediate memory score, with higher scores indicating better memory function [15].

**Fig. 1:**
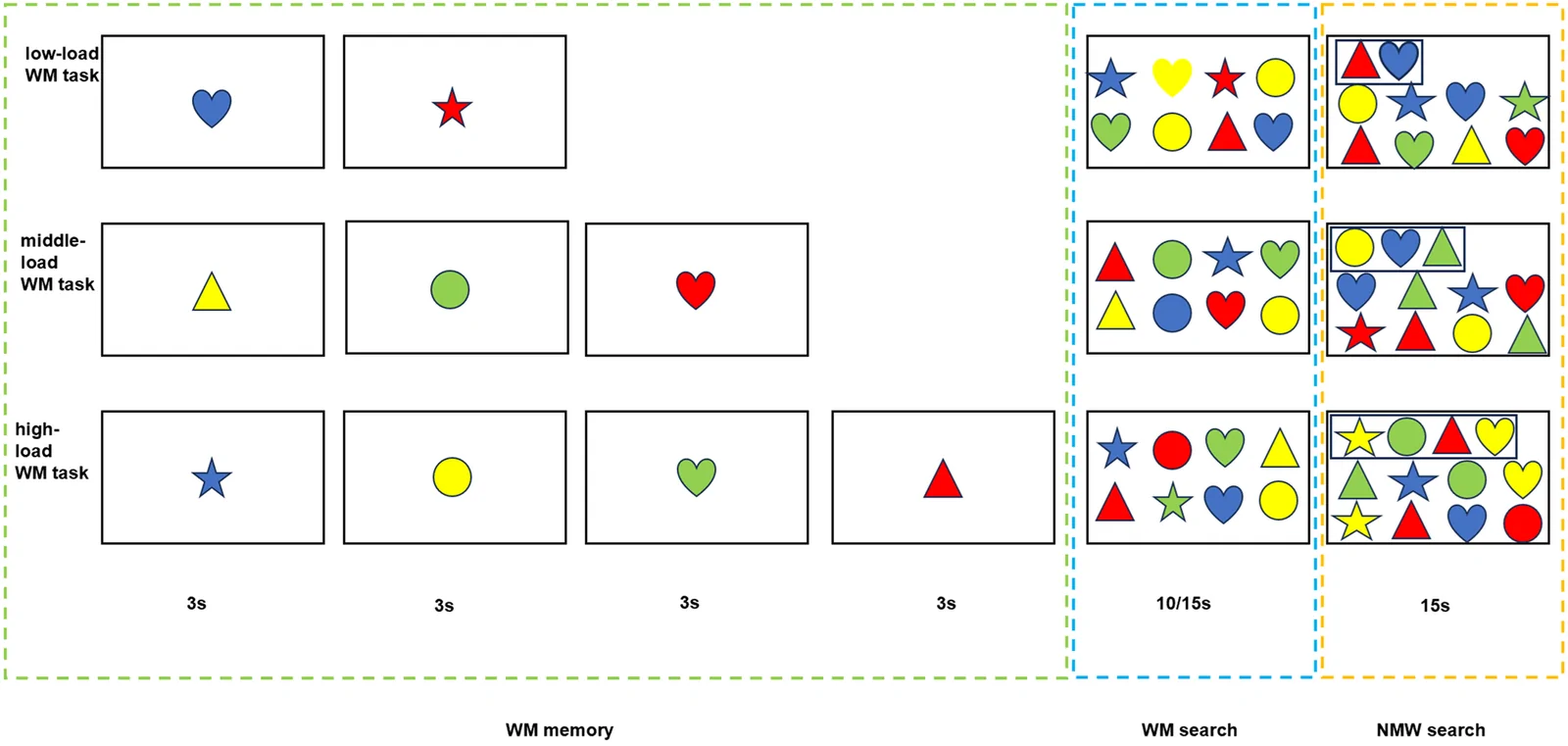
The protocol of the three WM-load tasks. Note: Low-load WM task: WM task total 19s = task cue 3s + Stage 1 (WM memory, one graph 3s, total 6s) + Stage 2 (WM search 10s), NWM task total 15s = Stage 3 (NWM search 15s). middle-load WM task: WM task total 22s = task cue 3s + Stage 1 (WM memory, one graph 3s, total 6s) + Stage 2 (WM search 10s), NWM task total 15s = Stage 3 (NWM search 15s). high-load WM task: WM task total 30s = task cue 3s + Stage 1 (WM memory, one graph 3s, total 6s) + Stage 2 (WM search 15s), NWM task total 15s = Stage 3 (NWM search 15s)

WM was evaluated using a task that included low-, middle-, and high-load conditions. Each session began with a 5-second welcome screen and a 5-second relaxation period, followed by four task sets (WM + Relax + Non-WM + Relax). The task involved random combinations of four shapes (circles, triangles, hearts, stars) and four colors (red, yellow, blue, green). In the low-load condition, Stage 1 required participants to remember the colors, shapes, and order of two sequentially displayed shapes (3 seconds each). Stage 2 involved recalling and clicking the shapes in the memorized order within 10 seconds, while Stage 3 was a non-WM task where participants clicked graphical cues without memorization for 15 seconds. The middle- and high-load conditions involved 3- and 4-stimuli memory tasks, respectively [16] (Fig. 1). Participants practiced once before the formal test to ensure understanding. During testing, they remained seated and avoided large movements to minimize interference.

Prior to the study, two experienced psychiatrists were trained to administer the assessments. Their inter-rater reliability, measured by correlation coefficients for HAMD-24, PSQI, and RBANS, exceeded 0.8 after repeated measures, ensuring consistency in evaluations. This rigorous training and validation process helped maintain the accuracy and reliability of the data collected throughout the study.

### fNIRS measurements

A 48-channel fNIRS system (NirScan, Danyang Huichuang Medical Equipment Co., Ltd., China) was used to record hemodynamic responses in the cerebral cortex of patients with MDD during the WM task. The system operated at a sampling frequency of 11 Hz and utilized near-infrared light at wavelengths of 730, 808, and 850 nm to measure changes in oxyhemoglobin (Oxy-Hb), deoxyhemoglobin (Deoxy-Hb), and total hemoglobin (Total-Hb) concentrations.

The fNIRS probes, comprising 15 light sources and 16 detectors, were positioned over the frontal and temporal regions according to the 10–20 international electroencephalogram (EEG) system. The inter-probe distance was fixed at 3 cm, with the bottom probe aligned along the Fp1-Fp2 line. The 48 channels were grouped into six regions of interest (ROIs) based on device coordinates: the right dorsolateral prefrontal cortex (R-DLPFC), left dorsolateral prefrontal cortex (L-DLPFC), right medial prefrontal cortex (R-mPFC), left medial prefrontal cortex (L-mPFC), right temporal lobe (R-TL), and left temporal lobe (L-TL). The specific channel-to-region mapping is illustrated in Supplementary materials 1. This setup allowed for precise localization and analysis of cortical hemodynamic activity during the WM task.

Two trained doctors conduct fNIRS assessments for each patient at a fixed time (from 10:00 to 11:00 AM). During the assessment, patients are guided to remain relaxed and avoid using electronic devices.”

### Data processing and analysis

The fNIRS data were processed using NirSpark analysis software. Motion artifacts and environmental noise were first corrected, followed by the removal of physiological noise using a 0.01–0.20 Hz bandpass filter. The Beer-Lambert law was then applied to convert the optical data into blood oxygen concentration values. Oxy-Hb changes were prioritized for analysis due to their stronger correlation with cortical activation and higher signal-to-noise ratio compared to Deoxy-Hb [17].

A 2-second pre-task interval was used as the baseline for normalization. The average Oxy-Hb activation changes were calculated for the WM task, with durations of 10 seconds, 10 seconds, and 15 seconds for the low-, medium-, and high-load conditions, respectively, as well as for the non-working memory (NWM) task (15 seconds) in each channel. Within each ROI, the Oxy-Hb concentration changes were averaged across all channels to derive the mean values for the WM task. This approach ensured a robust and standardized analysis of cortical hemodynamic responses during the task.

### Statistical analysis

The sociodemographic characteristics, clinical variables, accuracy rates, and Oxy-Hb activation during the WM task were first described for all subjects. The normality of all variables was confirmed using the Kolmogorov-Smirnov one-sample test. Group differences between MDD patients with and without insomnia were analyzed using one-way ANOVA for continuous variables and the chi-square test (χ^2^ test) for categorical variables, with Bonferroni correction applied for multiple comparisons. In order to adjust for the effects of HAMD, partial correlation analysis correlation coefficient was used to evaluate the relationship between working memory function and Oxy-Hb activation during the WM task in both groups. Additionally, multiple linear regression analyses were conducted in MDD patients with insomnia to identify brain regions with the strongest association between Oxy-Hb activation and immediate memory. Statistical analyses were performed using SPSS 25.0, with a two-tailed *p* < 0.05 considered statistically significant.

## Results

### Demographic and basic descriptive data

**Fig. 2:**
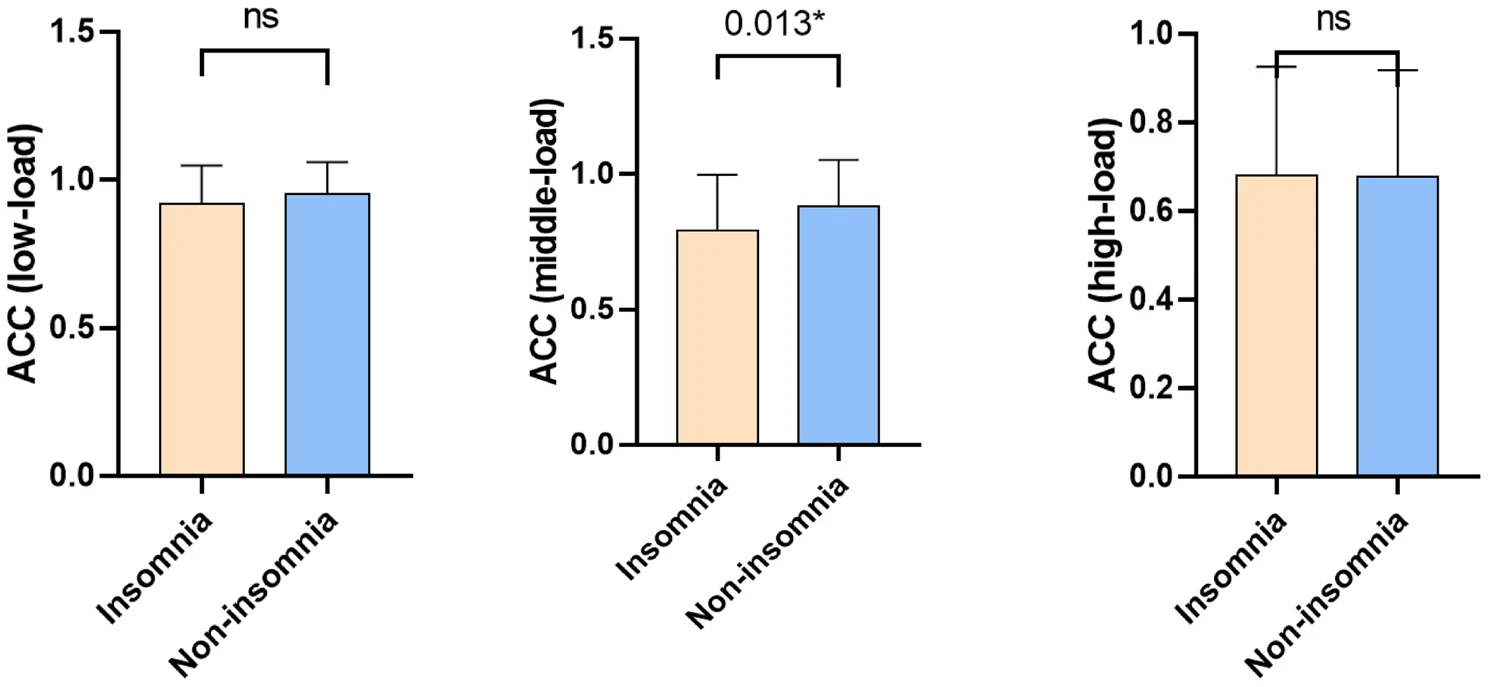
Accuracy rates during the WM task between MDD patients with or without insomnia. Note: ACC=Accuracy rate. Analysis of variance (ANOVA) and chi-square tests were used in Figure 2 to obtain significance and p values

No significant differences were observed between MDD patients with and without insomnia in age, education, gender, BMI, or and suicide (all *p* > 0.05), with significant differences in HAMD-24 scores (F = 4.818, *p* = 0.030), PSQI scores (F = 264.841, *p* < 0.001) and Immediate Memory (F = 6.733, *p* = 0.011). Additionally, while no significant differences were found in ACC for low- or high-load WM tasks (all *p* > 0.05), the two groups showed statistically significant differences in ACC for the middle-load WM task (F = 6902, *p* = 0.010) (Table 1 and Fig. 2).

**Table 1:** Demographic and clinical characteristics of MDD patients with or without insomnia

	Insomnia（*N* = 67）	Non-insomnia (*N* = 55)	F\x^2^	p-Value
Age(years)	30.84 ± 14.75	28.51 ± 11.83	0.896	0.346
Education(years)	11.87 ± 3.20	11.98 ± 2.40	0.050	0.824
BMI (kg/m^2^)	21.20 ± 3.84	21.86 ± 3.90	0.895	0.346
Gender(male/female)	17/50	22/33	2.971	0.085
Suicide（yes/no）	21/46	21/34	0.626	0.429
HAMD-24	25.87 ± 5.39	23.87 ± 4.46	4.818	0.030*
PSQI	14.49 ± 2.70	7.11 ± 2.22	264.841	＜0.001***
Immediate Memory	78.70 ± 16.66	86.69 ± 17.24	6.733	0.011*
**WM task**				
ACC (low-load)	0.92 ± 0.13	0.95 ± 0.11	2.392	0.142
ACC (middle -load)	0.79 ± 0.21	0.88 ± 0.17	6.354	0.013*
ACC (high-load)	0.68 ± 0.25	0.68 ± 0.24	0.008	0.930

Note: BMI = body mass index; HAMD-24 = 24-item Hamilton Depression Scale; PSQI = Pittsburgh sleep quality index; ACC = Accuracy rate; WM = working memory

The significance of bold emphases in the table is*p < 0.05; **p < 0.01; ***p < 0.001

Analysis of variance (ANOVA) and chi-square tests were used in Table 1 to obtain significance and *p* values

### Oxy-Hb values during WM task in MDD patients with and without insomnia

This study presents a comparison of the mean Oxy-Hb activation changes between MDD patients with and without insomnia. Patients with insomnia demonstrated significantly lower Oxy-Hb activation changes in the R-DLPFC (F = 6.555, *p* = 0.012) and L-DLPFC (F = 5.262, *p* = 0.024) during the middle-load WM task, as well as in the L-DLPFC (F = 4.449, *p* = 0.037) during the high-load WM task, compared to patients without insomnia. However, no significant differences in Oxy-Hb activation changes were observed between the two groups during the low-load WM task across all brain regions (all *p* > 0.05). These findings suggest that insomnia in MDD patients is associated with reduced prefrontal cortical activation, particularly under higher cognitive load conditions. (Table 2)

**Table 2:** Oxy-Hb values during the WM task between MDD patients with or without insomnia

	Insomnia（*N* = 67）	Non-insomnia (*N* = 55)	F	p-Value
**Oxy-Hb activation of low-load WM task**				
R-DLPFC	−0.0004±0.024	0.004 ± 0.026	1.055	0.306
L-DLPFC	−0.002±0.025	0.005 ± 0.027	1.981	0.162
R-TL	0.004 ± 0.037	0.010 ± 0.035	0.906	0.343
L-TL	0.008 ± 0.040	0.005 ± 0.038	0.203	0.653
R-mPFC	0.001 ± 0.029	0.005 ± 0.034	0.518	0.473
L-mPFC	−0.002±0.032	0.004 ± 0.034	0.891	0.347
**Oxy-Hb activation of middle-load WM task**				
R-DLPFC	−0.006±0.026	0.006 ± 0.026	6.555	0.012*
L-DLPFC	−0.005±0.021	0.007 ± 0.032	5.262	0.024*
R-TL	0.007 ± 0.048	−0.004±0.047	1.648	0.202
L-TL	0.009 ± 0.054	−0.001±0.043	1.299	0.257
R-mPFC	0.0005 ± 0.032	0.005 ± 0.033	0.579	0.448
L-mPFC	−0.003±0.042	−0.003±0.040	0.000	0.995
**Oxy-Hb activation of high-load WM task**				
R-DLPFC	−0.009±0.033	−0.004±0.042	0.560	0.456
L-DLPFC	−0.012±0.030	0.0002 ± 0.034	4.449	0.037*
R-TL	0.006 ± 0.066	−0.0006±0.055	0.368	0.545
L-TL	0.017 ± 0.059	0.007 ± 0.061	0.835	0.363
R-mPFC	−0.008±0.050	0.005 ± 0.049	1.889	0.172
L-mPFC	−0.012±0.049	−0.004±0.048	0.792	0.375

Note: WM = work memory; DLPFC = dorsolateral prefrontal cortex; mPFC = medial prefrontal cortex; TL = temporal lobe; *R* = right; L = left

The significance of bold emphases in the table is*p < 0.05; **p < 0.01; ***p < 0.001

Analysis of variance (ANOVA) were used in Table 2 to obtain significance and p values

### Relationship between working memory and Oxy-Hb values during the WM task in MDD patients with and without insomnia

**Fig. 3:**
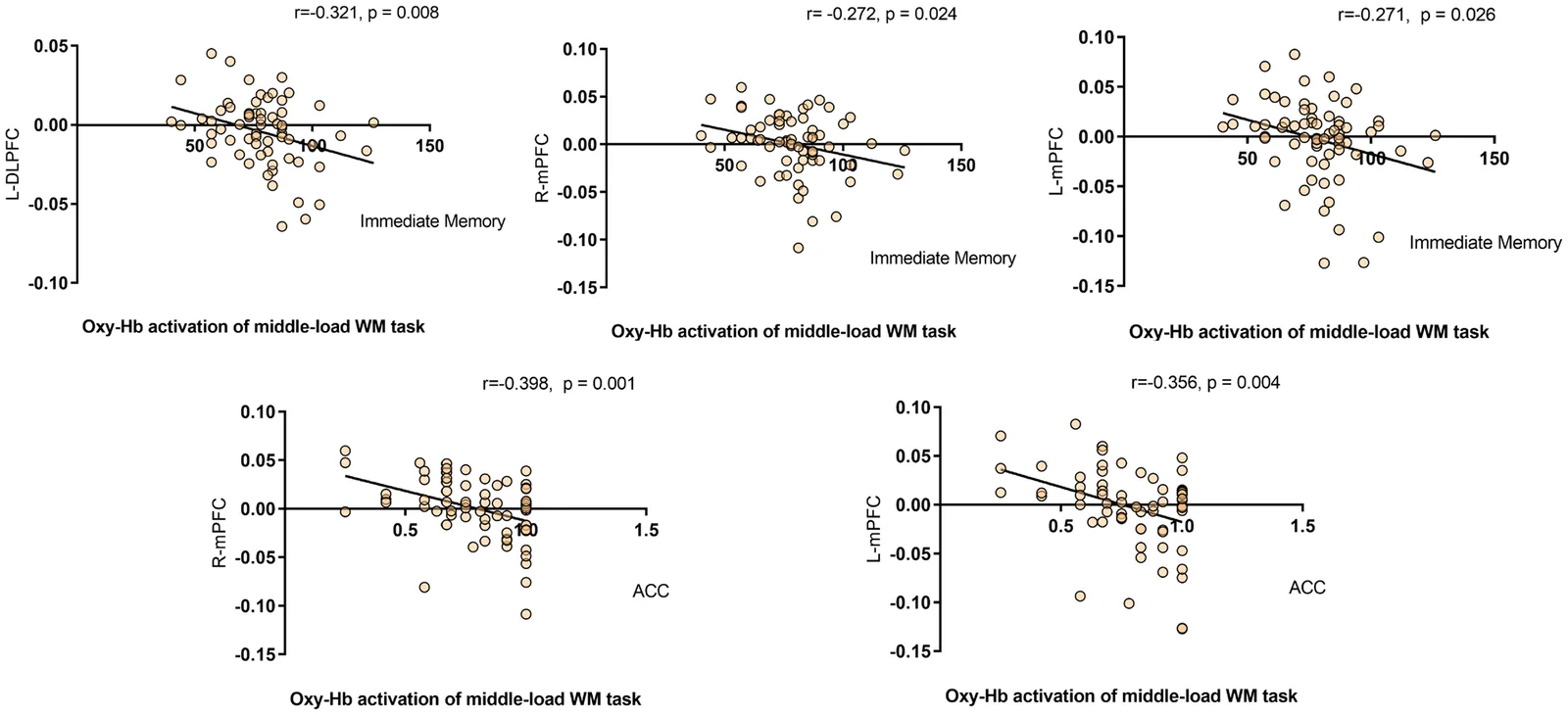
Relationship between WM and Oxy-Hb values during the task in MDD patients with insomnia. Note: DLPFC=dorsolateral prefrontal cortex; mPFC=medial prefrontal cortex; R=right; L=left; ACC=Accuracy rate, WM= working memory. Pearson’s correlation coefficients were used in Figure 3 to obtain significance and p values

Table 3 and Fig. 3 present the correlation between working memory function and mean changes in Oxy-Hb activation in MDD patients with insomnia after adjusting for the HAMD scale. Correlation analysis revealed that, during the middle-load WM task, the immediate memory subscale score was negatively correlated with Oxy-Hb activation in the L-DLPFC (*r* = −0.321, df = 65, *p* = 0.008), as well as the right (*r* = −0.272, df = 65, *p* = 0.024) and left (*r* = −0.271, df = 65, *p* = 0.026) medial prefrontal cortex (mPFC). Additionally, the ACC rate was negatively correlated with Oxy-Hb activation in the left (*r* = −0.356, df = 65, *p* = 0.004) and right (*r* = −0.398, df = 65, *p* = 0.001) mPFC. However, no significant correlations were observed between working memory performance and brain activation during the low- and high-load WM tasks. Further multiple linear regression analysis indicated that mean changes in Oxy-Hb activation in the L-DLPFC (B = −252.257, *t* = −2.753, *p* = 0.008, R^2^ change = 0.104) were independently associated with the immediate memory subscale score during the middle-load WM task.

**Table 3:** Relationship between WM and Oxy-Hb values during the task in MDD patients with insomnia

	IM (low-load)	IM (middle -load)	IM (high-load)	ACC (low-load)	ACC (middle-load)	ACC (high-load)
R-DLPFC	0.081	−0.161	−0.115	−0.072	−0.112	−0.096
L-DLPFC	−0.012	−0.321^**^	−0.059	−0.151	−0.173	−0.023
R-TL	−0.177	−0.160	−0.084	−0.029	−0.115	−0.134
L-TL	−0.102	−0.061	−0.052	0.084	−0.127	−0.032
R-mPFC	−0.079	−0.272^*^	−0.220	−0.212	−0.398^***^	−0.173
L-mPFC	−0.151	−0.271^*^	−0.198	−0.231	−0.356**	−0.124

Note: IM = Immediate Memory; ACC = Accuracy rate; DLPFC = dorsolateral prefrontal cortex; mPFC = medial prefrontal cortex; TL = temporal lobe; *R* = right; L = left

Significance levels of correlation coefficients are shown. *p < 0.05; **p < 0.01; ***p < 0.001

Pearson’s correlation coefficients were used in Table to obtain significance and *p* values

In contrast, for MDD patients without insomnia, mean changes in Oxy-Hb activation across all brain regions showed no significant correlations with the immediate memory subscale score or accuracy rate during the low-, middle-, or high-load WM tasks (all *p* > 0.05) (Table 4).

**Table 4:** Relationship between WM and Oxy-Hb values during the task in MDD patients with non-insomnia

	IM (low-load)	IM (middle -load)	IM (high-load)	ACC (low-load)	ACC (middle -load)	ACC (high-load)
R-DLPFC	−0.185	−0.069	0.113	−0.046	0.003	−0.015
L-DLPFC	−0.160	0.105	−0.085	−0.012	0.043	−0.022
R-TL	−0.128	0.019	−0.080	−0.042	−0.100	−0.057
L-TL	−0.143	0.216	0.051	−0.023	0.071	−0.022
R-mPFC	−0.132	0.021	−0.225	−0.031	−0.218	0.144
L-mPFC	−0.250	−0.020	−0.239	−0.054	−0.200	−0.013

Note: IM = Immediate Memory; ACC = Accuracy rate; DLPFC = dorsolateral prefrontal cortex; mPFC = medial prefrontal cortex; TL = temporal lobe; *R* = right; L = left

Significance levels of correlation coefficients are shown. *p < 0.05; **p < 0.01; ***p < 0.001

Pearson’s correlation coefficients were used in Table 4 to obtain significance and *p* values.

These findings suggest that, in MDD patients with insomnia, reduced prefrontal cortical activation during cognitive tasks is associated with poorer working memory performance, particularly under moderate cognitive load conditions.

## Discussion

This study represents the first investigation into WM function and prefrontal cortical activation patterns in MDD patients with and without insomnia using fNIRS during WM tasks. Our findings reveal three key insights: (1) MDD patients with insomnia exhibit greater WM impairment compared to those without insomnia; (2) reduced prefrontal Oxy-Hb activation, particularly in the DLPFC, is observed in the insomnia group during middle- and high-load WM tasks; and (3) WM performance is correlated with prefrontal Oxy-Hb activation specifically in MDD patients with insomnia. These results explore potential neurofunctional indicators linking insomnia with cognitive deficits in MDD and highlight the potential of fNIRS as a tool for screening different MDD subtypes.

The observed WM deficits in MDD patients with insomnia align with prior research demonstrating the detrimental effects of sleep disturbances on cognitive function, particularly memory processes [18]. Sleep disturbances, such as insomnia, are known to disrupt memory consolidation and retrieval, which are critical for effective WM performance. In healthy populations, sleep deprivation has been shown to impair memory encoding and retrieval, leading to fragmented memory processes. In MDD patients, insomnia may exacerbate these effects by impairing emotional regulation and increasing negative emotional states, further disrupting cognitive functions [19, 20]. Additionally, altered sleep architecture in MDD—characterized by increased rapid eye movement (REM) sleep and reduced slow-wave sleep (SWS)—may contribute to WM deficits [21, 22]. SWS plays a crucial role in memory consolidation and processing speed, and its reduction in MDD patients with insomnia may underlie the observed WM dysfunction. These findings underscore the importance of addressing insomnia in MDD patients as a modifiable factor that could improve cognitive outcomes.

Our study also revealed distinct prefrontal activation patterns between MDD patients with and without insomnia during WM tasks. During the middle-load WM task, MDD patients with insomnia exhibited inhibitory activation in the bilateral DLPFC, while those without insomnia showed excitatory activation in the same regions. A similar pattern was observed during the high-load WM task, with significantly weaker L-DLPFC activation in the insomnia group. However, no group differences in frontotemporal activation were observed during the low-load WM task, likely due to the task’s simplicity masking performance disparities. These findings suggest that insomnia-related cognitive deficits are more pronounced during complex WM tasks. The underlying mechanism may involve insomnia-related dysregulation of prefrontal cortical function, as sleep disturbances have been shown to reduce the connection between prefrontal and gray matter volume and impair neurophysiological processes critical for cognitive load management [23]. Impaired prefrontal synaptic plasticity and white matter integrity further compromise the prefrontal neural functions responsible for circadian sleep regulation, triggering sleep-onset difficulty, fragmented sleep and other sleep disturbances [24]. Meanwhile, as the core brain region underlying cognitive processing including working memory and immediate memory, structural and functional impairments of the prefrontal cortex simultaneously damage information storage and processing capacities, resulting in declines in general cognitive function and working memory performance [25]. Moreover, heightened arousal—a core feature of insomnia—has been linked to altered functional connectivity between the salience network (SN) and the DLPFC, with stronger SN-DLPFC coupling correlating with greater insomnia severity (measured by PSQI scores) [26]. These neurofunctional alterations likely contribute to the observed deficits in complex WM performance.

Another significant finding of this study is the negative correlation between Oxy-Hb concentrations in the left DLPFC and bilateral medial prefrontal cortex (mPFC) and immediate memory function in MDD patients with insomnia. Specifically, the Oxy-Hb concentration in the L-DLPFC was most strongly associated with immediate memory function. Additionally, during the middle-load WM task, the Oxy-Hb concentrations in the bilateral mPFC were negatively correlated with the accuracy rate in MDD patients with insomnia. These results suggest that impairments in working memory are closely related to reduced frontal cortex activation in MDD patients with insomnia. These findings are consistent with previous studies using VFT, which also reported negative correlations between memory function and Oxy-Hb concentrations in the L-DLPFC and left mPFC in MDD patients with insomnia(Yanli [17]). Mechanistically, this may reflect structural and functional alterations in the prefrontal cortex, such as reduced gray matter volume observed in insomnia-related MDD, which could impair synaptic efficiency and neural resource allocation during cognitive tasks [27, 28]. Additionally, diminished brain-derived neurotrophic factor (BDNF) levels—a key mediator of synaptic plasticity and neurogenesis—may exacerbate these deficits by disrupting neural circuits critical for memory encoding and retrieval [29]. To our knowledge, this is the first study to utilize fNIRS during a WM task paradigm to directly associate prefrontal hypoactivation with working memory impairments in MDD patients with insomnia, providing a neurofunctional framework for understanding cognitive decline in this comorbid population.

Despite these significant findings, our study has several limitations. Firstly, as a cross-sectional study, it is difficult to establish causality between insomnia, prefrontal activation, and WM deficits in MDD patients. Future longitudinal studies are needed to explore these relationships further. Secondly, the study was conducted in a single psychiatric hospital in Zhejiang Province, southern China, which may limit the generalizability of the findings to other regions or cultural backgrounds. Expanding the sample size and including participants from diverse geographical and cultural settings would enhance the external validity of the results. Third, the gender differences between the two groups in this was borderline were marginally significant, suggesting that the impact of sleep disorders on cognitive dysfunction may differ between male and female. Therefore, this issue should be further explored in future studies by balancing the gender ratio. Fourth, the study lacked serum or cerebrospinal fluid tests to identify potential molecular biomarkers of sleep and cognition (e.g., BDNF levels). Finally, our study did not control for medication use in MDD patients, which may influence brain activation patterns. Future research should account for the effects of antidepressants and other medications to better understand their impact on cognitive function and brain activation in this population.

## Conclusion

This study provides novel insights into the neurofunctional correlates of working memory deficits in MDD patients with insomnia. The reduced prefrontal activation observed in the insomnia group, particularly during complex WM tasks, suggests the potential of fNIRS as a diagnostic tool for screening different MDD subtypes. With the increasing availability of neuroimaging data such as fNIRS, artificial intelligence (AI) technologies hold promise in enhancing brain health promotion. Future work may incorporate machine learning algorithms to classify MDD subtypes (e.g., insomnia vs non-insomnia), predict cognitive outcomes based on prefrontal hemodynamic patterns, or assist in individualized treatment planning. By leveraging AI to decode complex brain-behavior relationships, we can move toward precision psychiatry that enables early detection and targeted intervention for cognitive impairment in MDD patients with insomnia.

## Electronic supplementary material

Below is the link to the electronic supplementary material.

Supplementary material 1

## Data Availability

Data available on request from the authors.

## Abbreviations

MDD: Major depressive disorder
WM: Working memory
fNIRS: Functional near-infrared spectroscopy
PSQI: Pittsburgh Sleep Quality Index
HAMD: Hamilton Depression Scale
RBANS: Repeatable Battery for the Assessment of Neuropsychological Status
DSM-5: Diagnostic and Statistical Manual of Mental Disorders, Fifth Edition
IM: Immediate Memory
Oxy-Hb: Oxyhemoglobin
Deoxy-Hb: Deoxyhemoglobin
Total-Hb: Total hemoglobin
EEG: Electroencephalogram
ROIs: Regions of interest
R-DLPFC: Right dorsolateral prefrontal cortex
L-DLPFC: Left dorsolateral prefrontal cortex
R-mPFC: Right medial prefrontal cortex
L-mPFC: Left medial prefrontal cortex
R-TL: Right temporal lobe
L-TL: Left temporal lobe
NWM: Non-working memory
REM: Rapid eye movement
SWS: Reduced slow-wave sleep
SN: Salience network
BDNF: Brain-derived neurotrophic factor
AI: Artificial intelligence
