# Supplementary material for: Investigating working memory and brain activation in major depressive disorder with and without insomnia: insights from functional near-infrared spectroscopy (fNIRS)
Source: BMC Psychiatry. 2026 Aug 19;26:897. doi: 10.1186/s12888-026-08471-6 (PMC13760743; doi:10.1186/s12888-026-08471-6)
Supplement: Supplementary file 1 [file 12888_2026_8471_MOESM1_ESM.pdf]

Supplementary materials 1: Schematic of arrangement of fNIRS channels.

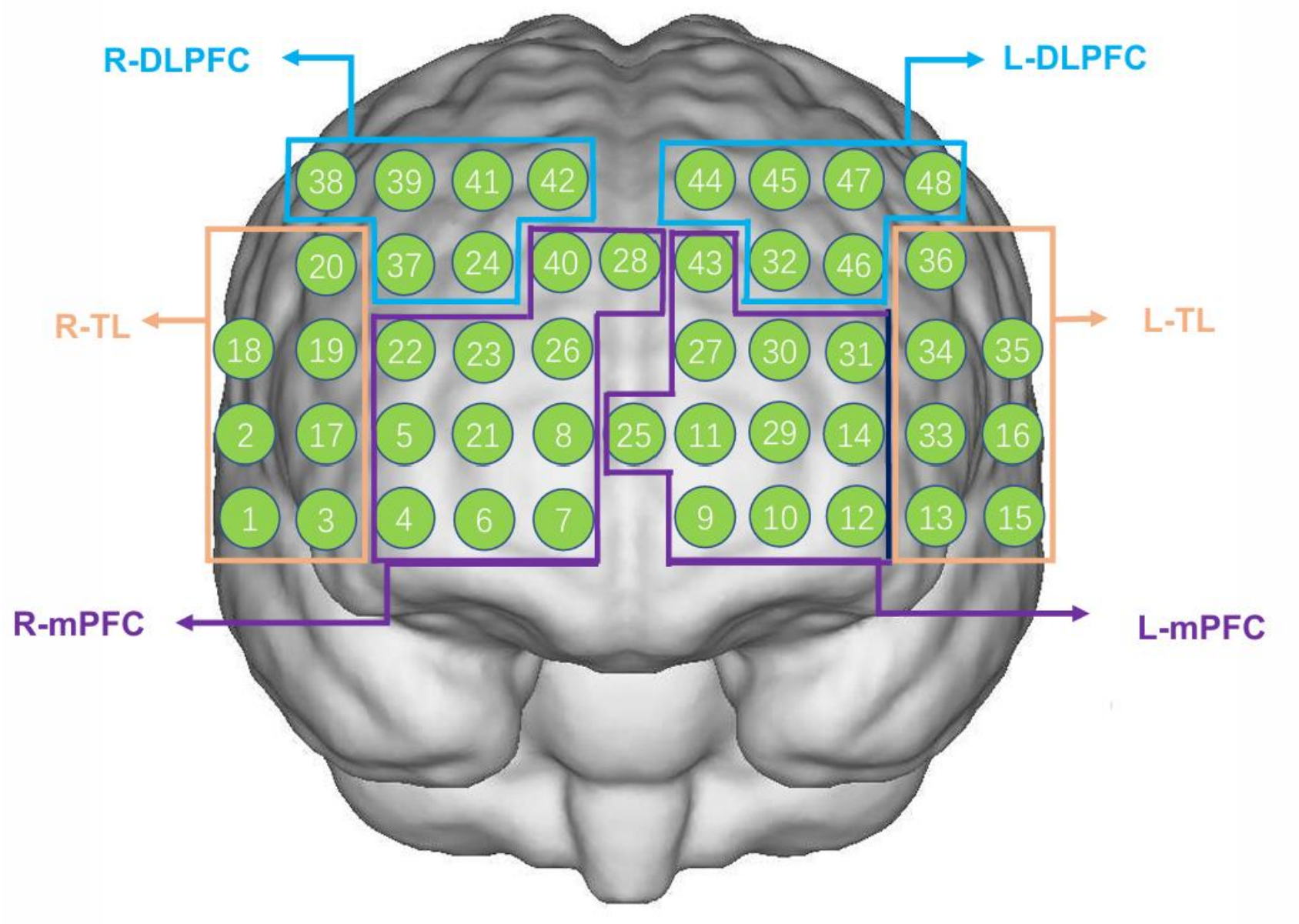

DLPFC=dorsolateral prefrontal cortex; mPFC=medial prefrontal cortex; TL=temporal lobe ; R=right; L=left.
